# Supplementary material for: Open neuroinformatics infrastructure ecosystem for federated multisite studies
Source: bioRxiv. 2026 May 5:2026.04.30.721944. Preprint. [Version 1] doi: 10.64898/2026.04.30.721944 (PMC13174541; doi:10.64898/2026.04.30.721944)
Supplement: Supplement 1 [file media-1.pdf]

	N	Age (years)	Male	Female
**Age task**				
PPMI	1082	64.4 ± 8.1	524 (48.4%)	558 (51.6%)
Calgary	58	71.4 ± 7.3	28 (48.3%)	30 (51.7%)
QPN	69	62.6 ± 11.9	26 (37.7%)	43 (62.3%)
ADNI	315	74.4 ± 6.1	158 (50.2%)	157 (49.8%)
PREVENT-AD	342	64.8 ± 5.9	98 (28.7%)	244 (71.3%)
COGTIPS	29	62.3 ± 9.8	62.3 ± 9.8	12 (41.4%)
**Cognitive decline task**				
PPMI	768	62.8 ± 9.4	484 (63.0%)	284 (37.0%)
Calgary	61	70.6 ± 6.5	41 (67.2%)	20 (32.8%)
QPN	41	64.1 ± 8.5	29 (70.7%)	12 (29.3%)
ADNI	617	72.3 ± 7.4	325 (52.7%)	292 (47.3%)
PREVENT-AD	342	64.8 ± 5.9	98 (28.7%)	244 (71.3%)
**Diagnosis task**				
PPMI	1908	63.7 ± 8.7	1044 (54.7%)	864 (45.3%)
Calgary	158	71.3 ± 6.7	94 (59.5%)	64 (40.5%)
QPN	290	64.8 ± 9.7	174 (60.0%)	116 (40.0%)
ADNI	1106	73.2 ± 7.1	586 (53.0%)	520 (47.0%)
COGTIPS	113	63.0 ± 8.1	68 (60.2%)	45 (39.8%)

Supplementary Table 1: Participant demographics. Age is given as mean ± standard deviation.

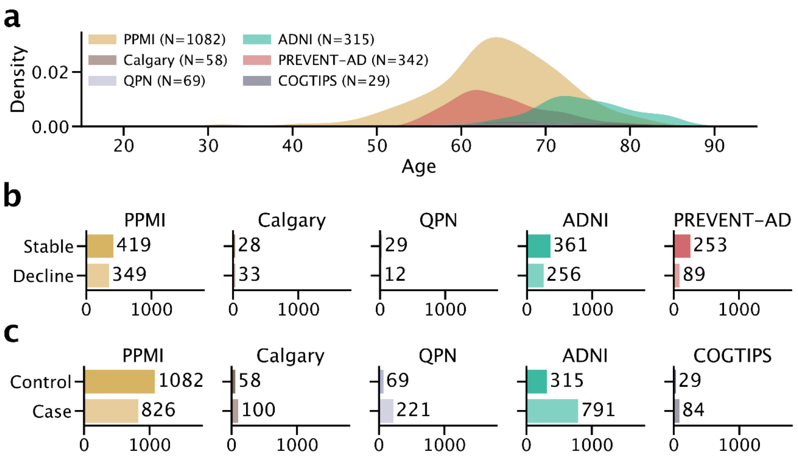

Supplementary Figure 1: Distributions of target variables in prediction tasks. a: Age. **b**: Cognitive decline. **c**: diagnosis.
